# Editorial: The link between obesity, type 2 diabetes, and mitochondria

**DOI:** 10.3389/fendo.2023.1229935

**Published:** 2023-06-20

**Authors:** Moulun Luo, Gaetano Santulli

**Affiliations:** ^1^ Division of Endocrinology, Department of Medicine, University of Arizona, Tucson, AZ, United States; ^2^ Center for Disparities in Diabetes, Obesity and Metabolism, University of Arizona Health Sciences, Tucson, AZ, United States; ^3^ Department of Medicine, Wilf Family Cardiovascular Research Institute, Fleischer Institute for Diabetes and Metabolism (FIDAM), Albert Einstein College of Medicine, New York, NY, United States; ^4^ Department of Molecular Pharmacology, Einstein-Mount Sinai Diabetes Research Center (ES-DRC), Einstein Institute for Aging Research, Institute for Neuroimmunology and Inflammation (INI), Albert Einstein College of Medicine, New York, NY, United States

**Keywords:** mitochondria, obesity, mitochondrial dysfunction, mitochondrial dynamics, type 2 diabetes mellitus, preventive medicine, cardiovascular risk, diabetes mellitus

The present Research Topic, entitled “*The Link between Obesity, Type 2 Diabetes, and Mitochondria*” aims at highlighting the functional role of the relationship linking mitochondria, obesity, and diabetes mellitus, including key mechanisms involved and the potential implications for therapeutic interventions. The relationship between obesity, type 2 diabetes (T2D), and mitochondria is multifaceted and complex; understanding this relationship can provide valuable insights into the prevention and management of T2D and obesity.

Obesity and T2D are major global health challenges with significant consequences for individuals and healthcare systems. The prevalence of both conditions has been steadily increasing over the past few decades (1). The underlying mechanisms connecting these two conditions, particularly the role of mitochondria, have gained considerable attention (2–4).

Mitochondria are vital organelles responsible for cellular energy production through oxidative phosphorylation. Obesity has been associated with mitochondrial dysfunction, including impaired mitochondrial biogenesis, reduced oxidative capacity, and increased oxidative stress. These alterations can lead to inefficient energy utilization, contributing to metabolic abnormalities observed in obesity. Mitochondrial dysfunction can negatively affect insulin signaling pathways. Impaired mitochondrial oxidative capacity can result in increased levels of reactive oxygen species (ROS), and activation of stress-related pathways, all of which interfere with insulin action. Consequently, a condition of reduced biological response to insulin in peripheral tissues (*i.e.* insulin resistance) develops (5–7). Rautenberg et al. elegantly describe the normal function and structure of mitochondria and highlight some of the key studies that demonstrate mitochondrial abnormalities in skeletal muscle of volunteers with T2D and obesity. Additionally, they explain epigenetic modifications in the context of insulin resistance and mitochondrial abnormalities, emphasizing mitochondrial DNA methylation.

Mitochondrial dynamics, including fusion, fission, and mitophagy, can play crucial roles in maintaining mitochondrial quality and function (8–10). Dysregulation of these processes in obesity can further exacerbate mitochondrial dysfunction and impair cellular metabolism. Emerging evidence suggests that modulating mitochondrial dynamics may have therapeutic potential in preventing or reversing metabolic disorders associated with T2D and obesity (11, 12). Mitochondrial metabolism controls glucose-stimulated insulin secretion (GSIS) by ATP production, redox signaling, and Ca^2+^ handling in pancreatic β cells (13–15). Pacifici et al. demonstrate that peroxiredoxin 6 (*Prdx6*), an antioxidant enzyme with both peroxidase and phospholipase A2 activity, finely controls mitochondrial homeostasis and plays a pivotal role in the regulation of glucose-stimulated insulin release.

Oxidative stress is also a fundamental component of the pathogenesis of diabetic cardiomyopathy: ROS generation in cardiomyocytes starts a vicious circle, resulting in mitochondrial DNA damage, post-translational modification of proteins, lipid peroxidation, further production of ROS, eventually culminating in inflammation, cardiac hypertrophy, interstitial fibrosis, and cardiac dysfunction. The main signaling pathways related to oxidative stress in diabetic cardiomyopathy are examined in a comprehensive review (Peng et al.).

Thermogenic adipocytes possess a promising approach to combat obesity with its capability promoting energy metabolism. In this sense, Luo et al. demonstrate that deleting G protein-coupled receptor 30 (GPR30), a membrane-associated estrogen receptor, drives the activation of mitochondrial uncoupling respiration to induce adipose thermogenesis in female mice; indeed, GPR30 deficiency enhances beige adipocyte differentiation in white adipose tissue. This novel mechanism could potentially lead to novel therapeutic strategies to prevent the development of obesity and obesity related metabolic diseases.

Hence, targeting mitochondrial dysfunction and dynamics represents a promising avenue for the development of therapeutic interventions for T2D and obesity (**Figure 1**). Strategies such as exercise, calorie restriction, pharmacological agents, and nutraceuticals have shown potential in improving mitochondrial function and insulin sensitivity (16). Further research is needed to elucidate the precise molecular mechanisms and identify effective interventions that can improve mitochondrial function and mitigate the metabolic consequences of T2D and obesity. Additionally, novel approaches, including mitochondrial-targeted antioxidants, are being explored for their potential to restore mitochondrial function and mitigate metabolic abnormalities.

**Figure 1:**
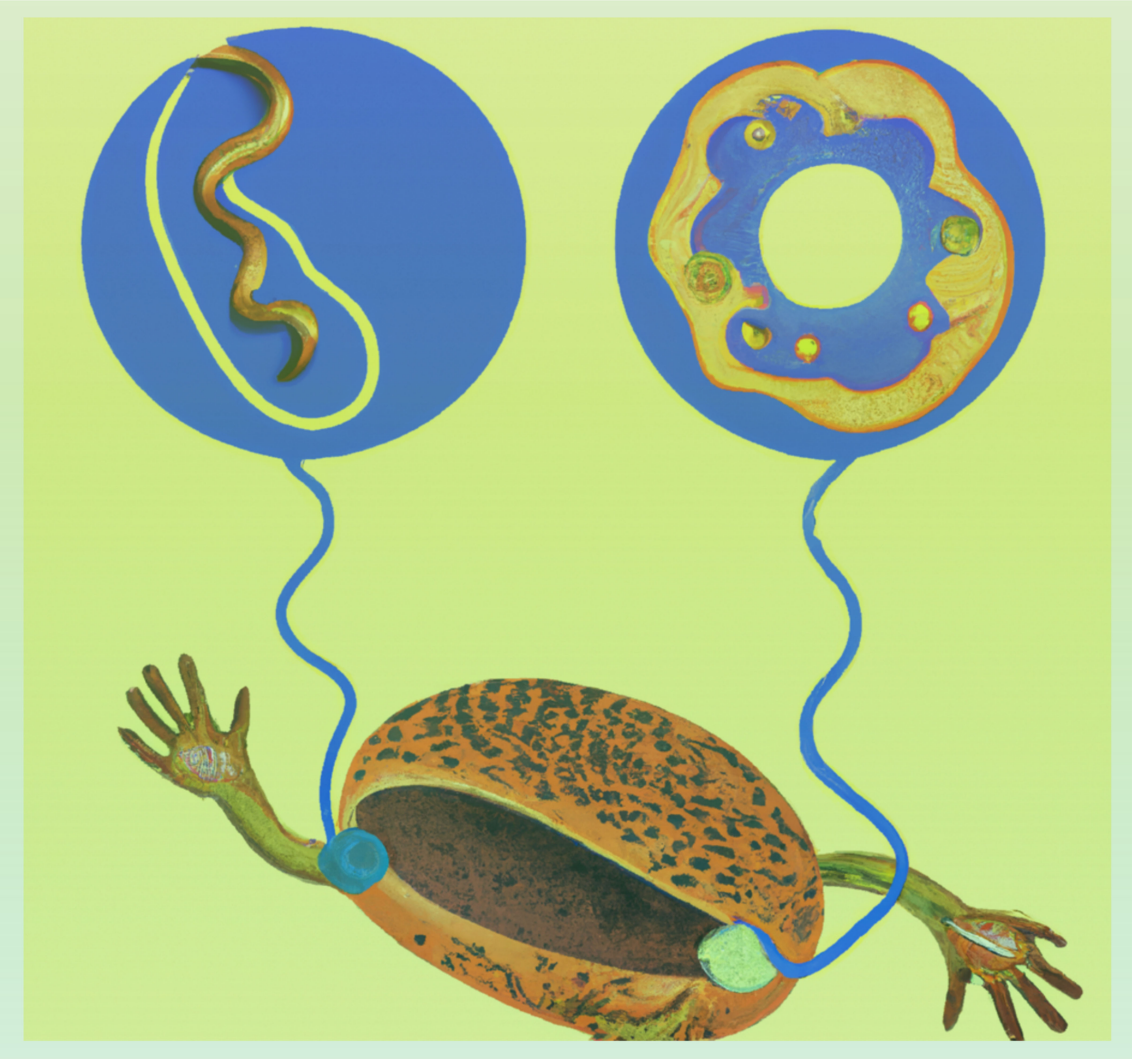
Artistic representation linking mitochondria, obesity, and diabetes.

In conclusion, exploring the link between obesity, T2D, and mitochondria enhances our understanding of the pathophysiology of these conditions. This knowledge can inform the development of novel therapeutic approaches to prevent and manage T2D and obesity, potentially reducing the burden of these diseases on individuals and healthcare systems.

## Author contributions

All authors listed have made a substantial, direct, and intellectual contribution to the work and approved it for publication.

## References

[1] Varzideh F Kansakar U Jankauskas SS Gambardella J Santulli G . Cardiovascular endocrinology: evolving concepts and updated epidemiology of relevant diseases. Front Endocrinol (Lausanne) (2021) 12:772876. doi: 10.3389/fendo.2021.772876 34675888 PMC8524081

[2] Kolb H Kempf K Rohling M Lenzen-Schulte M Schloot NC Martin S . Ketone bodies: from enemy to friend and guardian angel. BMC Med (2021) 19(1):313. doi: 10.1186/s12916-021-02185-0 34879839 PMC8656040

[3] Yin L Luo M Wang R Ye J Wang X . Mitochondria in sex hormone-induced disorder of energy metabolism in males and females. Front Endocrinol (Lausanne) (2021) 12:749451. doi: 10.3389/fendo.2021.749451 34987473 PMC8721233

[4] Cheng H Gang X He G Liu Y Wang Y Zhao X . The molecular mechanisms underlying mitochondria-associated endoplasmic reticulum membrane-induced insulin resistance. Front Endocrinol (Lausanne) (2020) 11:592129. doi: 10.3389/fendo.2020.592129 33329397 PMC7719781

[5] Zapata-Bustos R Finlayson J Langlais PR Coletta DK Luo M Grandjean D . Altered transcription factor expression responses to exercise in insulin resistance. Front Physiol (2021) 12:649461. doi: 10.3389/fphys.2021.649461 33897458 PMC8058368

[6] Shu Y Wu X Wang J Ma X Li H Xiang Y . Associations of dietary inflammatory index with prediabetes and insulin resistance. Front Endocrinol (Lausanne) (2022) 13:820932. doi: 10.3389/fendo.2022.820932 35250879 PMC8892213

[7] Mone P Morgante M Pansini A Jankauskas SS Rizzo M Lombardi A . Effects of insulin resistance on mitochondrial (dys)function. Atherosclerosis (2022) 341:52–4. doi: 10.1016/j.atherosclerosis.2021.11.026 PMC8943707 34903382

[8] Yu R Lendahl U Nister M Zhao J . Regulation of mammalian mitochondrial dynamics: opportunities and challenges. Front Endocrinol (Lausanne) (2020) 11:374. doi: 10.3389/fendo.2020.00374 32595603 PMC7300174

[9] Gambardella J Jankauskas S Kansakar U Varzideh F Avvisato R Prevete N . Ketone bodies rescue mitochondrial dysfunction via epigenetic remodeling. JACC Basic Transl Sci (2023). 10.1016/j.jacbts.2023.03.014 PMC10543927 37791311

[10] Poderoso C Filippi BM Maloberti PM Franco MC . Editorial: mitochondrial dynamics in endocrine physiology and disease. Front Endocrinol (Lausanne) (2022) 13:844842. doi: 10.3389/fendo.2022.844842 35250891 PMC8889580

[11] Haigh JL New LE Filippi BM . Mitochondrial dynamics in the brain are associated with feeding, glucose homeostasis, and whole-body metabolism. Front Endocrinol (Lausanne) (2020) 11:580879. doi: 10.3389/fendo.2020.580879 33240218 PMC7680879

[12] Dai W Jiang L . Dysregulated mitochondrial dynamics and metabolism in obesity, diabetes, and cancer. Front Endocrinol (Lausanne) (2019) 10:570. doi: 10.3389/fendo.2019.00570 31551926 PMC6734166

[13] Lombardi A Trimarco B Iaccarino G Santulli G . Impaired mitochondrial calcium uptake caused by tacrolimus underlies beta-cell failure. Cell Commun Signal (2017) 15(1):47. doi: 10.1186/s12964-017-0203-0 29132395 PMC5684747

[14] Schultz J Warkus J Wolke C Waterstradt R Baltrusch S . MiD51 is important for maintaining mitochondrial health in pancreatic islet and MIN6 cells. Front Endocrinol (Lausanne) (2020) 11:232. doi: 10.3389/fendo.2020.00232 32411091 PMC7198722

[15] Lombardi A Gambardella J Du XL Sorriento D Mauro M Iaccarino G . Sirolimus induces depletion of intracellular calcium stores and mitochondrial dysfunction in pancreatic beta cells. Sci Rep (2017) 7(1):15823. doi: 10.1038/s41598-017-15283-y 29158477 PMC5696524

[16] Krako Jakovljevic N Pavlovic K Jotic A Lalic K Stoiljkovic M Lukic L . Targeting mitochondria in diabetes. Int J Mol Sci (2021) 22(12):6642. doi: 10.3390/ijms22126642 34205752 PMC8233932

